# Predicting Depression in Adolescents Using Mobile and Wearable Sensors: Multimodal Machine Learning–Based Exploratory Study

**DOI:** 10.2196/35807

**Published:** 2022-06-24

**Authors:** Tahsin Mullick, Ana Radovic, Sam Shaaban, Afsaneh Doryab

**Affiliations:** 1 Department of Engineering Systems and Environment University of Virginia Charlottesville, VA United States; 2 Department of Pediatrics University of Pittsburgh Pittsburgh, PA United States; 3 NuRelm Pittsburgh, PA United States

**Keywords:** adolescent, depression, uHealth, machine learning, mobile phone

## Abstract

**Background:**

Depression levels in adolescents have trended upward over the past several years. According to a 2020 survey by the National Survey on Drug Use and Health, 4.1 million US adolescents have experienced at least one major depressive episode. This number constitutes approximately 16% of adolescents aged 12 to 17 years. However, only 32.3% of adolescents received some form of specialized or nonspecialized treatment. Identifying worsening symptoms earlier using mobile and wearable sensors may lead to earlier intervention. Most studies on predicting depression using sensor-based data are geared toward the adult population. Very few studies look into predicting depression in adolescents.

**Objective:**

The aim of our work was to study passively sensed data from adolescents with depression and investigate the predictive capabilities of 2 machine learning approaches to predict depression scores and change in depression levels in adolescents. This work also provided an in-depth analysis of sensor features that serve as key indicators of change in depressive symptoms and the effect of variation of data samples on model accuracy levels.

**Methods:**

This study included 55 adolescents with symptoms of depression aged 12 to 17 years. Each participant was passively monitored through smartphone sensors and Fitbit wearable devices for 24 weeks. Passive sensors collected call, conversation, location, and heart rate information daily. Following data preprocessing, 67% (37/55) of the participants in the aggregated data set were analyzed. Weekly Patient Health Questionnaire-9 surveys answered by participants served as the ground truth. We applied regression-based approaches to predict the Patient Health Questionnaire-9 depression score and change in depression severity. These approaches were consolidated using universal and personalized modeling strategies. The universal strategies consisted of Leave One Participant Out and Leave Week X Out. The personalized strategy models were based on Accumulated Weeks and Leave One Week One User Instance Out. Linear and nonlinear machine learning algorithms were trained to model the data.

**Results:**

We observed that personalized approaches performed better on adolescent depression prediction compared with universal approaches. The best models were able to predict depression score and weekly change in depression level with root mean squared errors of 2.83 and 3.21, respectively, following the Accumulated Weeks personalized modeling strategy. Our feature importance investigation showed that the contribution of screen-, call-, and location-based features influenced optimal models and were predictive of adolescent depression.

**Conclusions:**

This study provides insight into the feasibility of using passively sensed data for predicting adolescent depression. We demonstrated prediction capabilities in terms of depression score and change in depression level. The prediction results revealed that personalized models performed better on adolescents than universal approaches. Feature importance provided a better understanding of depression and sensor data. Our findings can help in the development of advanced adolescent depression predictions.

## Introduction

### Background

According to the World Health Organization, half of all mental health conditions start at the age of 14 years, but most cases are undetected and untreated. Among mental health conditions, depression is one of the leading causes of illness and disability among adolescents [1], the most likely mental illness to be a risk factor for suicide [2], the second leading cause of death among US adolescents [3], and among the top causes of death in adolescents worldwide [4].

Major depressive disorder, more commonly termed *depression*, can be defined as a medical disorder that results in negative feelings in a person’s thoughts or actions. The effects of depression are both emotional and physical [5]. The sources of depression are varied and include biochemical changes, genetics, personality traits, and environmental factors [5]. Depression has a combination of effects that play a role in its diagnosis, such as alteration in mood, negative self-image, self-punitive desires, vegetative changes, and physiological changes such as activity retardation or agitation [6].

Depression is difficult to monitor or regulate in adolescents as part of the Diagnostic and Statistical Manual of Mental Disorders, Fifth Edition, diagnosis [7] includes not only depressive symptoms but also irritability, which may be difficult to distinguish from typical adolescent behavior. As an internalizing disorder that is expressed more through thoughts and not actions, worsening depressive symptoms can be more difficult for others such as parents or caregivers to identify [8]. Adolescents also report using cognitive coping strategies far less often than adults [9]. In a study on adolescent mental health literacy, it was found that <50% of adolescents were able to identify depression [10]. Although earlier intervention on symptom worsening improves outcomes in depression, adolescents themselves and their caregivers not being able to identify these symptoms serves as a barrier [11]. This indicates a strong need for interventions that can assist adolescents and their caregivers in monitoring the symptoms of depression earlier.

The result of not addressing depression can extend into adulthood, impairing both physical and mental health and limiting future employment opportunities and the potential to lead satisfied lives [12]. With the increased use of screening tools such as the Patient Health Questionnaire-9 (PHQ-9), mental health clinicians and primary care providers can more efficiently screen for depression. However, screening does not always lead to a substantial increase in treatment engagement [13]. Measurement-based care [14] or using these validated screening tools as recurring to identify, monitor, and treat depressive symptoms results in improved outcomes for patients with depression by identifying and intervening earlier on worsening nonresponsive symptoms or their treatment [15].

The success of validated screening tools has provided mental health clinicians and primary care providers with better assessment tools for symptom severity and, especially with the ability to embed these tools in electronic health records, more frequent monitoring may result in earlier intervention and improved care. Unfortunately, constant monitoring of depression symptomatology is still far from reality. With the advent of mobile phones, fitness trackers, and their inbuilt sensors, this can be made possible. Our goal is to look closely into adolescent depression through the eyes of passively sensed data and evaluate machine learning (ML) approaches that offer predictive capabilities.

By exploring approaches to adolescent depression data, we want to enable the future development of apps geared toward the continuous monitoring of patients experiencing depression and allow clinicians, adolescents, and their parents the opportunity to take preventive or earlier actions.

This study was aimed at using passively sensed data to generate predictions on depression levels and change in depression levels. The predictions took on both universal and personalized modeling approaches. We then determined key contextual features that affected our ML models. Finally, we presented how the performance of personalized models changed over time and across data samples.

### Related Work

Related work in this section takes an inverse pyramid approach to describe the state of the art in mobile sensing for health apps and then focuses on the impact in the space of mental health.

#### Mobile-Based Sensing for Health Apps

Mobile sensing has been an active research area in health apps. A number of studies [16-18] have analyzed areas of cardiovascular health and sensed participant heart rate and heart rate variability with the help of mobile camera sensors. Areas of study such as sleep have benefited from mobile sensing by using sensors to detect sleep quality and sleep states using ML [19]. Further studies on sleep have explored both supervised and unsupervised approaches to detect sleep variation in contextual settings [20-22]**.** Mobile sensors such as accelerometers, gyroscopes, and GPSs have been used to model human behavior and cognition through contextualized feature extraction [23,24]. Studies on overall health and well-being have combined the aforementioned sensing capabilities to help promote general health. For example, the use of health apps to monitor human behavior through sleep, physical activity, and social interaction [25] has been found to show improvement in behavior patterns. Another example of general well-being [26] generates an index as a medium of feedback for improving health through exercise-based goal setting. All of the aforementioned studies have shown the efficacy of using mobile sensing to predict or diagnose health-related changes. The next subsection delves into how mobile sensing is changing mental health.

#### Mobile Sensing in Mental Health Apps

Mobile sensing–based mental health studies have been conducted in the areas of bipolar disorder [27], schizophrenia [28], anxiety [29,30], stress [31,32], and depression [33-39]. These studies have shown that mobile sensing can play an integral role in detecting and predicting mental health–related problems. Daily mood, physical activity, and social communication tracking of participants helped predict symptoms of bipolar relapse [27]. This was achieved using random coefficient methods to analyze the relationship between phone-based data and the rating of manic and depressive symptoms. Schizophrenia is another mental condition in which passive sensing has demonstrated predictive capability by showing the relationships between tracked features as indicators of schizophrenia [28]. The study used bivariate analysis and tree-based methods to perform ecological momentary assessment scores. Depression and anxiety in college students were studied, in particular the effect of stress and self-esteem, using the tool of causal networks derived from time-series sensor data [29]. These data helped in understanding the causal relationship between anxiety, depression, and stress. Anxiety regulation using wearable devices was explored through false feedback of slow heart rate [30] and was found to be beneficial for helping control anxiety symptoms. Researchers have been successful in tracking physiological changes during stress using voice sensing across different acoustic environments and individuals [31]. Patients undergoing chemotherapy were studied using passively sensed data. This exploratory study used instruments of random forest classifiers showing a strong correlation between sedentary behavior, less time spent in light physical activity, and other factors such as longer onscreen time and app interactions [32]. All of these studies provide sufficient evidence to consider passively sensed data as an effective method to track mental health, which provides support for our approach in this study.

One of the first studies to use mobile phones for depression used GPS data to track participant mobility [33]. The study provided evidence of a correlation between location-based data and depressive mood. In addition to GPS, phone use has been another feature to exhibit a strong relationship with depression severity [34]**.** The study extracted features such as phone use frequency and duration along with GPS-based features such as location variance and normalized entropy to show the correlation with depression. Behavior in people with depression has also been investigated by monitoring additional features such as sleep and social interaction through smartphones [35]. Multimodal features were gradually introduced into the research sphere to derive a contextual filtering of features that detected depression in college students and showed that multimodal feature information could outperform unimodal features [36]. This study used association rule mining to choose features and applied standard ML to detect depression, showing the merit in using multimodal features. Detecting depression is dependent on the approach used; analyzing the problem from the perspective of longitudinal data and exploring changes in depressive symptoms were shown to generate good accuracy [37]. The work in the latter study is closely related to our endeavor and serves as an inspiration. Collaborative filtering-based study is yet another approach that has shown promise in using personalized models to derive better predictions [38]. Our study also proposes 2 personalized strategies to model individual participants using ML.

Narrowing down to adolescent depression studies, we present some existing works in the literature and later explain their differences from our work in Table 1. Studies on adolescent depression have been primarily survey-based and social sentiment– and feasibility-centric [40-42]. The work in the study by Cao et al [39] closely relates to our aim of detecting depression in adolescents. However, their study had a smaller sample size and used both parent and adolescent inputs and was also more reliant on participant feedback. The differences between the studies highlighted and our work are further elaborated on in the *Discussion* section.

In this study, we first investigated the feasibility of universal and personalized ML modeling strategies to predict adolescent depression scores and change in depression levels. We then identified features that were more predictive of adolescents’ depression during the ML process. Finally, we studied how missingness of data affected model performance along with understanding how much data were required for our models to perform over a predetermined threshold.

Our findings revealed that a regression-based predictive modeling approach was able to capture more granular changes in depression scores. We also showed that personalized strategies were more effective predictors compared with universal strategies. The performance of personalized models did not improve with increase in the weeks of data and, instead of a steady increase in model performance with increase in data, we experienced fluctuations in the results. In an attempt to explain this phenomenon, we performed additional studies that separated our participants into 2 pools. The pools were generated based on the SD of the depression scores. Our results showed that the pool with a small SD in depression score was more accurately modeled in comparison with the higher-SD pool.

**Table 1:** Papers on adolescent mental health prediction and how our work differs from the existing work.

Paper	Study aim	Methods	Results	Difference from our work
Cao et al [39]	Investigated the effectiveness of smartphone apps useful in evaluating and monitoring depression symptoms in a clinically depressed adolescent population compared with psychometric instruments (PHQ-9^a^, HAM-D^b^, and HAM-A^c^); 13 participants aged 12 to 17 years	Used self-evaluation of adolescents and parents with smartphone data to improve predictions of PHQ-9 scores; used the SOLVD app installed only on Android phones; used only linear regressor and support vector regressor with polynomial kernel	Correlation between mood averaged over a 2-week period and biweekly psychometric score from PHQ-9, HAM-D, and HAM-A; combining self-evaluation from both parents and children along with smartphone sensor data resulted in PHQ-9 score prediction accuracy	Our work does not depend on self-evaluation by adolescents and parents to help improve predictions; instead, we consider a system where our reliance is exclusively on the captured sensor values to make predictions of PHQ-9 scores. We used universal and personalized modeling strategies with multiple machine learning algorithms.
Maharjan et al [43]	StandStrong app used to assess feasibility and acceptability of sensing technologies for maternal depression treatment in low-resource settings for mothers aged between 15 and 25 years	They explored possible explanations for differences in successful data collection by time of day and sensor type along with description of qualitative results to illuminate these differences	The study mainly identified concerns related to technological barriers in passively sensed data collection.	The study was based on passively sensed data collection. It did not perform predictive modeling. The aim was to assess how well the app performed in data collection and the hurdles encountered therein. The study had 11 participants with depression with a mix of young and older participants, whereas our study was focused on adolescents, and all participants had been diagnosed with some form of depression.
MacLeod et al [44]	Explored whether passively collected smartphone sensor data can be used to predict internalizing symptoms among youths in Canada; participants aged between 10 and 21 years	Self-reports of anxiety, depression, and attention-deficit hyperactivity disorder collected; N=122 for 2 weeks of passively sensed data; CES-DC^d^ and SCARED^e^ anxiety assessments were used	Depressive symptoms correlated with time spent stationary, less mobility, higher light intensity during the night, and fewer outgoing calls. Anxiety correlated with less time spent stationary, greater mobility, and more time on-screen. Adding passively collected smartphone data to prediction models of internalizing symptoms significantly improved their fit.	This work was primarily focused on establishing correlations between self-reports. The study used passive sensor data to perform linear regressor model fitting for predictions of the CES-DC and SCARED values. Nonlinear modeling approaches were not considered, whereas we have explored and produced better results.

^a^PHQ-9: Patient Health Questionnaire-9.

^b^HAM-D: Hamilton Depression Rating Scale.

^c^HAM-A: Hamilton Anxiety Rating Scale.

^d^CES-DC: Center for Epidemiological Studies Depression Scale for Children.

^e^SCARED: Screen for Child Anxiety Related Disorders.

## Methods

### Data Collection

#### Recruitment and Participant Breakdown

Adolescents aged 12 to 17.99 years and their parents were recruited from psychiatric clinics at the University of Pittsburgh Medical Center Western Psychiatric Hospital serving depressed and suicidal youth, an adolescent and young adult medicine clinic seeing youth for primary and subspecialty services, as well as through the University of Pittsburgh research registry. A total of 114 adolescents expressed an interest in this study. Of these 114 adolescents, 94 (82.5%) completed a screening assessment, and 31 (27.2%) were screened out because of minimal symptoms of depression (PHQ-9 score [45] of <5), no self-reported previous diagnosis of depression, not having a smartphone, and age restrictions. A total of 57 adolescents and their parents consented to the study, of whom 55 (96%) completed a baseline assessment and were entered into the study. The aggregated data set after exploratory data analysis (EDA) and initial cleaning consisted of 67% (37/55) of the participants. The reduction in participant number was due to sensor issues and irregular syncing that constituted missing data and the dropping out of some participants in between the study. The data for each participant were collected over a period of 24 weeks.

Passively sensed data from mobile phones were collected using the AWARE app [24], which logs relevant sensor data and harnesses those data within the device. It was installed on the participants’ phones and set up to record the sensor information in the desired sampling frequencies. We collected data from multiple sensors, including calls, conversations, location, Wi-Fi, and screen use. Features were classified into event-based features, which included phone use, calling, and conversational recording, and time series–based features, comprising Wi-Fi and GPS-based location. We used Fitbit Inspire HR (software version 1.84.5) to collect heart rate, sleep, and steps. Sensor data from GPS and Wi-Fi were collected at a frequency of 10 minutes. The Fitbit features were collected every minute and accumulated daily. The data collected from both AWARE and Fitbit were uploaded to the cloud and then hosted in a database for cleaning and further processing.

The AWARE passive sensing data and Fitbit were, on average, 69.11% and 32.36% complete, respectively. Missing Fitbit data were attributed to less than expected adherence to wearing the Fitbit because of several reasons, including forgetting to wear it, fatigue, rash (recurred in 1/55, 2% of the participants even after the band was changed), and the need to charge the device. The data collection process was approved by the University of Pittsburgh Institutional Review Board.

Weekly PHQ-9 surveys were sent over the 24 weeks, and the adolescents completed 69.01% (873/1265) of the weekly surveys on average, respectively. The PHQ-9 is an evaluative questionnaire used to assess depression severity. The PHQ-9 has been used effectively in multiple studies related to depression [38,39]. The questionnaire consists of a set of 9 questions with scores between 0 and 3. This results in an overall score range between 0 and 27. For the purpose of our study, this was our choice ground truth owing to its strength in categorizing depression severity levels and its effectiveness in yielding responses from participants when administered remotely [46,47]. The scores are divided into levels based on depression severity and allow for easier interpretability by clinicians, parents, and adolescents [45].

#### Descriptive Statistics of Collected Participant Data

**Figure 1:**
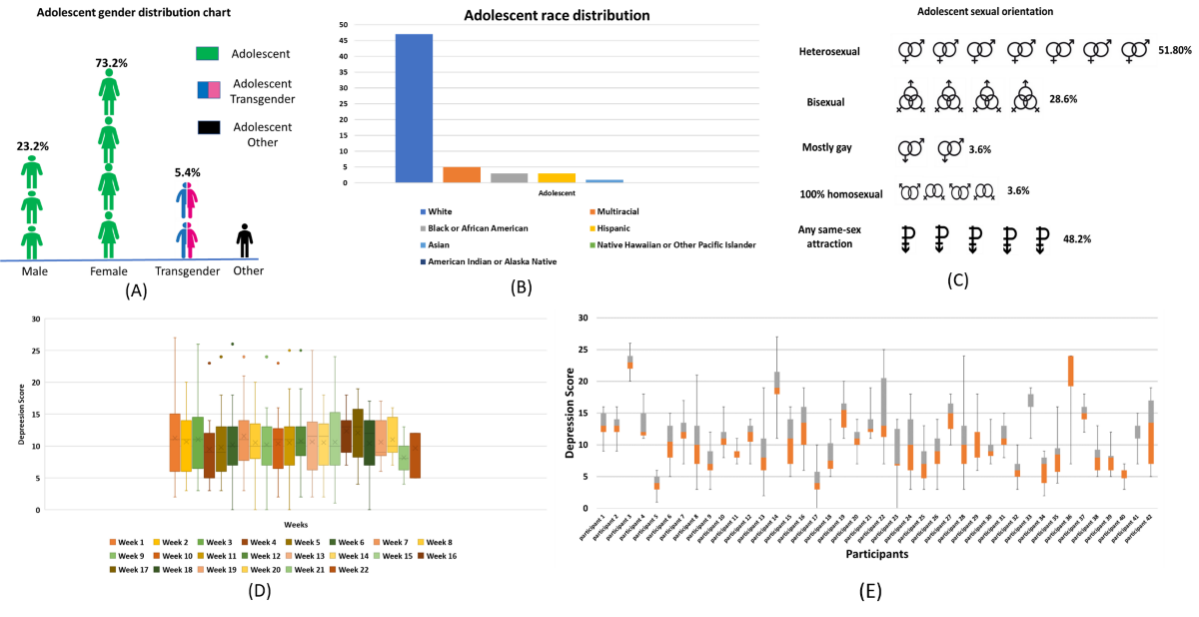
Demographic statistics: (A) gender distribution, (B) race distribution of the adolescents, (C) sexual orientation, (D) depression score distribution for each week of observation, and (E) depression score distribution for each participant.

The adolescent sample included participants aged 12 to 17.99 years, with an average age of 15.5 years. Most of the sample was White (47/56, 84%), with 16% (9/55) of the individuals representing a minority population. There was variability in gender, with approximately 73% (41/56) of the adolescent sample identifying as female, 23% (13/56) identifying as male, and 9% (3/56) identifying as transgender or other. The demographic statistics are provided in Figure 1 associated with the data collected.

Figure 1 also contains box plots of depression scores based on weeks (bottom left) and depression scores based on the participants’ box plots (bottom right). The depression score versus participants box plot presents the variation in depression scores across participants. The data set comprised 507 data points. The PHQ-9 scores ranged from a minimum of 0 to a maximum of 27. The mean PHQ-9 score was 11.21 (SD 5.23). For depression score versus weeks, we observed a mean PHQ-9 score of 10.63 (SD 4.92), and the minimum and maximum values were similar to those of the participant plot. The PHQ-9 scores are also expressed in the form of levels of depression: minimal (0-4), mild (5-9), moderate (10-14), moderately severe (15-19), and severe (20-27). The distribution of depression levels according to the number of participants was as follows: minimal depression (12/55, 22%), mild depression (26/55, 47%), moderate depression (31/55, 56%), moderately severe depression (21/55, 38%), and severe depression (5/55, 9%). There were rare occurrences of participants traversing up to 4 levels of depression over the course of their time in the study. It is also important to mention that, owing to data limitations and survey completion rate, 5% (3/55) of the participants maintained a single level of depression in the data set. The observations also revealed that most participants fluctuated between 2 levels of depression.

### Feature Extraction

The collected sensor data were passed to the Reproducible Analysis Pipeline for Data Streams framework [25] for feature extraction. The data set retained 66 features, including calls, conversations, locations, screen, Wi-Fi, heart rate, sleep, and steps. The data were then compiled into an aggregated data set and used as input for our ML modeling operations.

**Figure 2:**
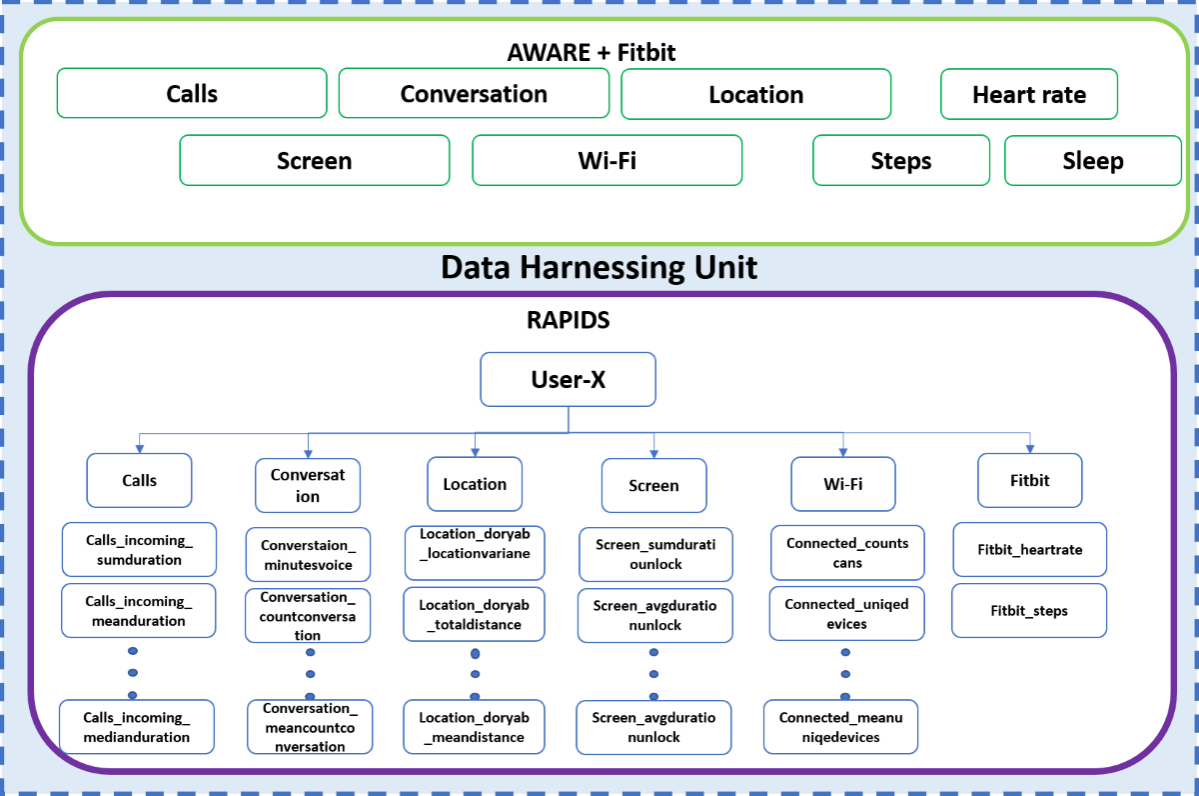
Feature extraction.

The data set was in a 2D tabular format for the application of our supervised modeling approaches populated with the survey results from the PHQ-9 weekly surveys to serve as the ground truth. To match the weekly ground truth depression score, we aggregated our features into daily and then weekly values. Figure 2 shows the combined harnessing framework comprising AWARE, Fitbit, and Reproducible Analysis Pipeline for Data Streams. Each sensor-based feature set was used to extract a range of features.

### ML Modeling

#### Overview

**Figure 3:**
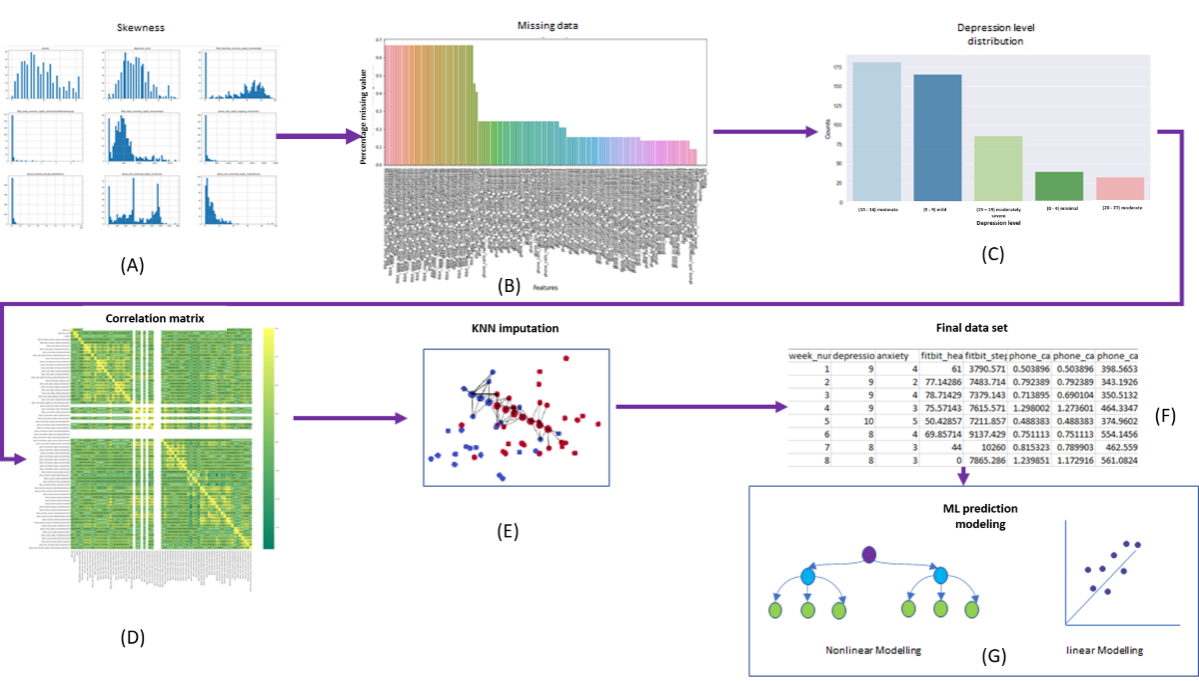
Machine learning (ML) pipeline comprising exploratory data analysis that includes (A) check for skewness of data, (B) missing value assessment, (C) check of depression level distribution, (D) generation of correlation matrix and removal of features that are highly correlated, (E) k-nearest neighbors (KNN)-based missing value imputation, (F) aggregated data set creation, and (G) nonlinear and linear ML modeling of data.

The data processing pipeline started with extensive EDA to check for skewness and filter missing data. This step was followed by the calculation of the Pearson correlation values for our feature set and the removal of highly correlated features. On the basis of our EDA, we set thresholds for missing data and adopted a robust imputation strategy such as the k-nearest neighbors, which is effective in handling multivariate time-series data. Our final data set consisted of 507 data points with 61 features, which represented 37 participants owing to high data sparsity. An illustration of the EDA and final data set generation is presented in Figure 3.

The ML phase after the data preprocessing can be segmented into a model-fitting stage and a cross-validation (CV) stage.

In the model-fitting stage, we applied both the depression score prediction and change in depression level prediction approaches. This model involved passing the feature sets through linear and nonlinear ML algorithms. The linear algorithms included Least Absolute Shrinkage and Selection Operator and elastic net. Nonlinear modeling included tree-based algorithms such as random forest; decision trees; and ensemble methods such as AdaBoost, extra trees, gradient boosting, and XGBoost.

The CV stage was responsible for the train-test splitting of data. This stage was also designed to consider universal and personalized modeling strategies. The universal strategies ensured that the modeling was based on the sample population data splits. The personalized strategies modeled based on individual data train-test splits. These strategies will be further elaborated on in the following subsection.

#### Prediction of Depression Score

**Figure 4:**
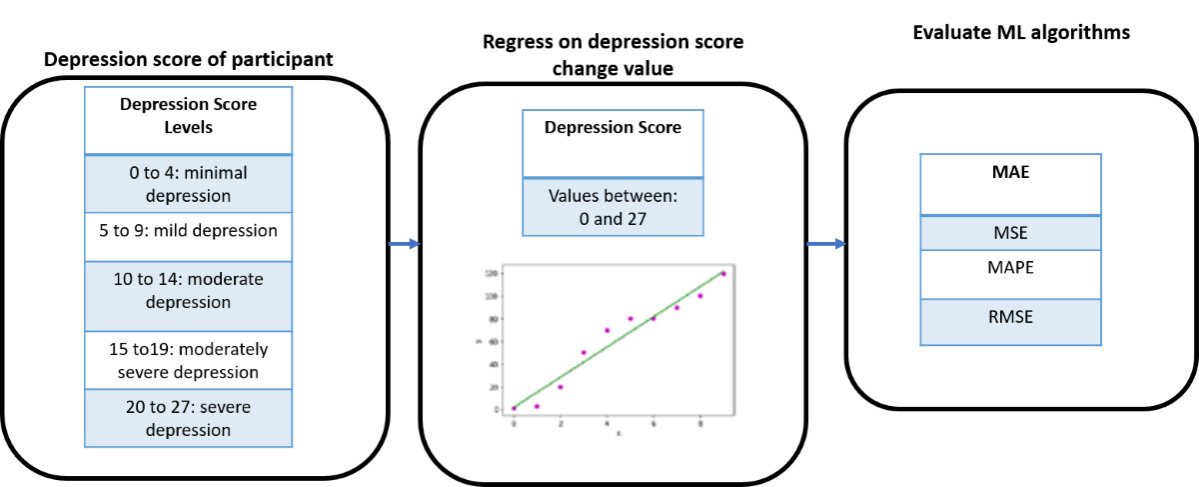
Depression score prediction approach. MAE: mean absolute error; MAPE: mean absolute percentage error; ML: machine learning; MSE: mean squared error; RMSE: root mean squared error.

To predict depression score, we used linear and nonlinear regression-based ML algorithms, as shown in Figure 4. The algorithms included Least Absolute Shrinkage and Selection Operator, elastic net, random forest, AdaBoost, extra trees, gradient boosting, and XGBoost for regression. The features extracted were used as input based on sensor combinations. The ML algorithms modeled on the data output predictions of the depression score. The model was based on universal and personalized modeling strategies. The models were evaluated based on mean absolute error (MAE), mean squared error (MSE), root MSE (RMSE), and mean absolute percentage error (MAPE).

#### Prediction of Change in Depression Level

**Figure 5:**
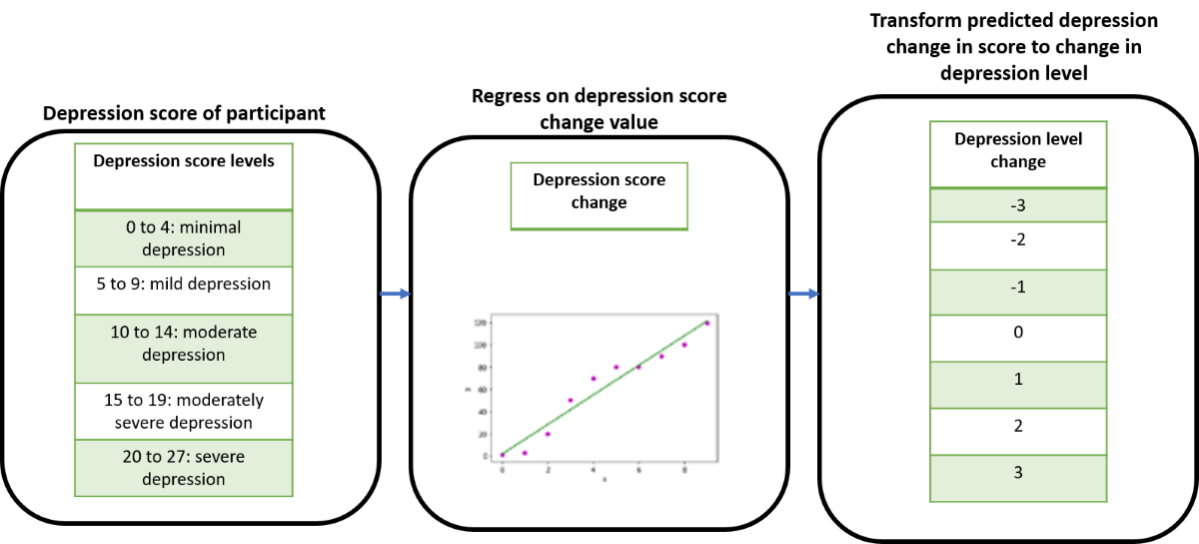
Machine learning approach for predicting change in depression level.

The prediction of change in depression level used the feature set combinations as input. The ML algorithms regressed on the feature data to predict the change in depression score and is shown in Figure 5. The change in depression level was then derived from the predicted change in depression score. This was a regression modeling approach with MAE, MSE, RMSE, and MAPE as evaluation metrics.

As mentioned previously, there were 5 depression levels. A jump to a level above (positive change) or a level below (negative change) was considered a change in level. The magnitude of the change was determined by the number of jumps seen in participant depression levels. On the basis of this idea, there were 9 changes in levels: positive changes (1, 2, 3, and 4), negative changes (−1, −2, −3, and –4), and no change (0). The change in depression level observed in our data fell within the range of −3 to 3. The change in depression scores was mapped to these 7 changes in depression levels. The establishment of levels helps in the better interpretation of depression changes by health care providers and aligns with standard medical diagnostics [45]**.**

### CV Strategy

**Figure 6:**
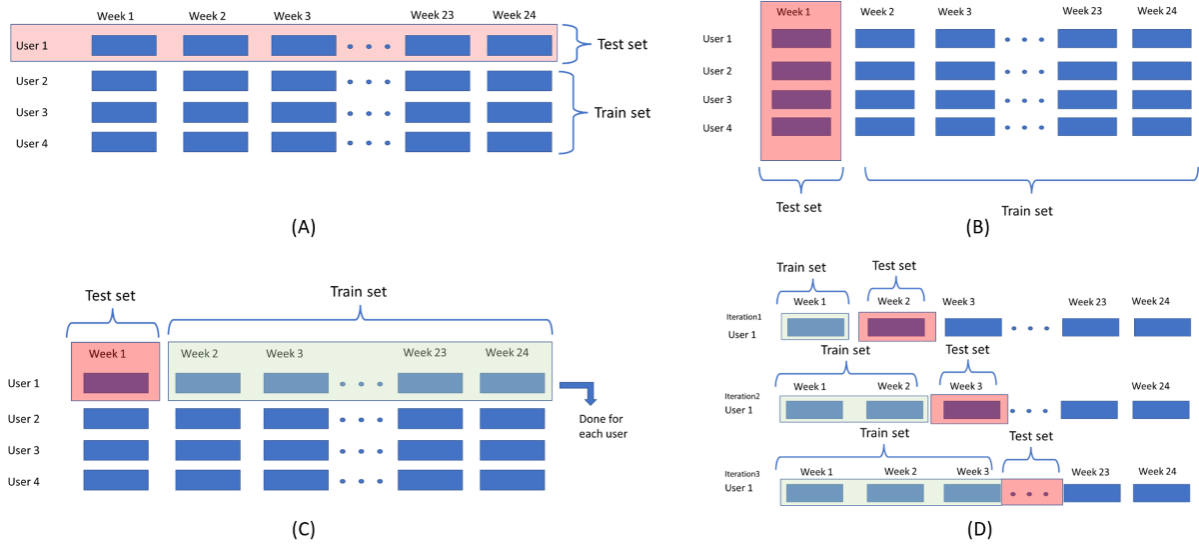
Cross-validation strategies: (A) Leave One User Out, (B) Leave Week X Out, (C) Leave One Week One User Instance, and (D) Accumulated Weeks.

We used multiple variations of the leave-one-out CV as presented in Figure 6. These strategies were designed to accommodate both personalization and generalization of the trained models.

#### Leave One Participant Out

In this strategy, we held out a single participant for validation and trained the model on the other participants. This strategy reflects the cold start case where a new user starts using the health app. This is a generalized approach to model fitting that takes advantage of the existing data set participants.

#### Leave Week X Out

In Leave Week X Out, we held out a given week for all participants and trained on the rest of the weeks. This strategy evaluates the impact of time-specific segments of data on the prediction. The training phase captures the similarity and variation of the data during different weeks to build the models. This too is categorized as a general modeling strategy to detect patterns in weekly depressive behavior.

#### Accumulate Weeks

A sliding window approach was followed in this CV strategy where, for each participant, the model was built with data from weeks *t* to *t*+*n* and tested on week *t*+*n*+1. This strategy examines the feasibility of the personalized ML models using data from individual users and evaluates the impact of longer-term data on prediction accuracy.

#### Leave One Week One User Instance Out

Here, we trained the models on all the weeks of a participant leaving one of their weeks for testing. This was done for all participants. This method also evaluates the feasibility of the personalized models using each individual user’s data on a week-by-week basis without considering the temporal and historical trend.

#### Baseline Performance

The idea of a baseline was to establish a reference for our accuracy levels. In this study, we operated with a naïve random baseline for our depression score approach and a majority baseline for the depression level change approach. This baseline analysis was carried out for all the CV strategies.

### Feature Set–Based Detailed Modeling

#### Overview

As shown in Figure 1, we used 6 major feature sets: Fitbit, calls, conversations, location, screen, and Wi-Fi. The aggregated data set was used to generate 63 individual data sets that comprised all possible combinations of these 6 feature sets. This approach was used to determine the most effective feature set combination for a specific modeling strategy. Each data set was passed through the modeling strategies and ML algorithms. The process of generating models for the various combinations of data sets is outlined in Textbox 1.

**Process to generate models for the various combinations of data sets.**

**Process of model generation**

- Data set generation: from the aggregated data set that is inclusive of all the feature sets, we generated all possible combinations of the feature sets, including 1-feature sets, 2-feature sets, 3-feature sets, 4-feature sets, 5-feature sets, and 6-feature sets.
- Depression score and change in depression level: we used the Patient Health Questionnaire-9 scores as the ground truth for depression score prediction. To predict the change in depression level, the ground truth was the actual transitions between the depression levels of the participants.
- Machine learning modeling: the derived data sets were all passed through both the universal and personalized modeling strategies.
- Model selection: for depression score prediction and change in depression level both following regression-based approaches, we evaluated and selected the best models based on mean absolute error, mean squared error, root mean squared error, and mean absolute percentage error precision.
- Feature importance: the models that performed best were further analyzed, and their feature importance was calculated. For each combination of sensors and their respective analyses under universal and personalized modeling strategies, the top 10 features were determined. This list of the top 10 features across the combinations was then converted into a frequency chart to help understand the features that had predictive capability.

#### Feature Importance Calculation

The feature importance in this study was calculated by observing the decrease in node impurity of our tree-based models, which included random forest, AdaBoost, and XGBoost. The impurity for regression tree-based modeling was determined by calculating the variance reduction of the model owing to a feature. Training the tree-based models allowed us to evaluate the contribution of each feature in decreasing the weighted impurity. This decrease in impurity was averaged over the ensemble of trees trained.

### Ethics Approval

We obtained approval for this study from the University of Pittsburgh Human Research Protections Office (STUDY18120176). After the participants showed interest in study involvement, they were screened based on the study criteria. The inclusion criteria were that the adolescents be aged 12 to 17.99 years, own an Android or iOS smartphone with access to a data plan, score ≥5 on the PHQ-9 consistent with at least mild symptoms, self-report a previous diagnosis of depression, understand English, and currently reside in the United States. The exclusion criteria were that the adolescents could not have current active suicidal ideation (thoughts with an intent to act on them), a history of a suicide attempt without having received mental health treatment, or a physical deformity or medical reason preventing them from wearing an activity tracker, or be simultaneously participating in a different research study using AWARE. Adolescents meeting the study criteria were offered study participation and, thereafter, if interested, provided their verbal assent, and their parents provided permission. A copy of the consent materials was emailed to all participants for review beforehand.

## Results

### Overview

In this section, we present the performance of our approaches in predicting depression score and change in depression level in adolescents. The results are the mean values of our runs with the respective approaches. We further show the features that played the most significant role in predicting outcomes for change in depression level. We then report the effect of adding incremental weekly data on the accuracy of depression level prediction. We analyzed the data using both universal and personalized modeling strategies. The study also assessed the impact of missing data on personalized modeling performance. Finally, we conclude this section with a comparative study of classic time-series modeling and a personalized ML model.

### Prediction of Depression Score

To understand how sensor features can help in predicting adolescents’ depression, we applied regression-based ML algorithms to predict depression scores. The model was compared with a random baseline, and we tested all possible combinations of sensor features and ML algorithms. The evaluation metrics selected were MAE, MSE, MAPE, and RMSE. In particular, we paid close attention to MAE and RMSE. As shown in Table 2, nonlinear algorithms such as the decision trees and AdaBoost performed best. Overall, the personalized models outperformed the universal models in all metrics, in particular MAE and RMSE. The best set of performance metrics recorded was for the Accumulate Weeks personalized strategy. The most optimal model used a 4-feature combination that consisted of Fitbit, calls, screen, and location-based feature sets (MAE=2.39, MSE=10.28, RMSE=2.83, MAPE=0.27). The best results from the personalized models were derived from feature sets that had location, calls, and screen in the combination. This also shows that adding more features does not necessarily yield better results. An RMSE in the range of 2 indicates that our model was able to predict depression scores within 2 scores of test cases.

**Figure 7:**
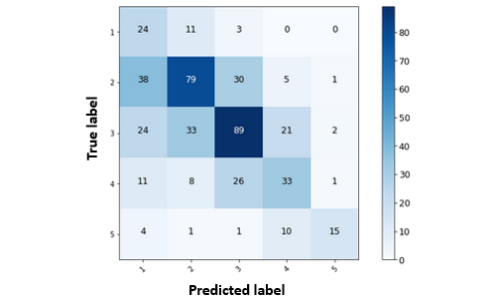
Confusion matrix of depression levels based on depression score predictions.

The results of the regression analysis of depression scores can also be interpreted as levels of depression. This was achieved by segmenting our predictions into intervals of PHQ-9 scores adhering to the established strategy [45]. The confusion matrix of depression levels in Figure 7 was derived based on the depression score predictions to provide more insight. We see that the model is able to best predict levels 2 and 3 most of the time with 89 and 79 correct labels, whereas the other levels, such as 1, 4, and 5, appear to have been predicted 24, 33, and 15 times, respectively. These results support our ground truth distribution, where mild (level 2) and moderate (level 3) depression accounted for most of the samples, followed by moderately severe (level 4), minimal (level 1), and severe (level 5) categorizations.

**Table 2:** Depression score regression results^a^.

	LOPO^b^	LWXO^c^	ACCU^d^	LOWOU^e^
MAE^f^ (SD)	4.46 (0.62)	3.43 (0.70)	2.39 (0.10)	2.53 (0.10)
MSE^g^ (SD)	30.74 (0.41)	19.0 (0.39)	10.28 (0.21)	11.89 (0.25)
MAPE^h^ (SD)	0.55 (0.65)	0.42 (0.52)	0.27 (0.15)	0.29 (0.20)
RMSE^i^ (SD)	5.07 (0.71)	4.31 (0.65)	2.83 (0.11)	2.53 (0.17)
Feature set	Fitbit, calls, conversation, screen, location, and Wi-Fi	Calls, conversation, screen, location, and Wi-Fi	Fitbit, calls, screen, and location	Fitbit, calls, conversation, screen, location, and Wi-Fi
ML^j^ algorithm	AdaBoost	Random forest	XGBoost	Random forest

^a^The values presented display evaluation metrics for depression score regression models. The best-performing machine learning models were AdaBoost, random forest, and XGBoost.

^b^LOPO: Leave One Participant Out.

^c^LWXO: Leave Week X Out.

^d^ACCU: Accumulate Weeks.

^e^LOWOU: Leave One Week One User Instance Out.

^f^MAE: mean absolute error.

^g^MSE: mean squared error.

^h^MAPE: mean absolute percentage error.

^i^RMSE: root mean squared error.

^j^ML: machine learning.

### Prediction of Change in Depression Level

Table 3 presents the results of predicting change in depression score. In this approach, the change in depression score was calculated between participant weeks as per depression score_time_ **–** depression score_time–1_. The best-performing models with the lowest MAE were the personalized models Accumulate Weeks (MAE=3.21, MSE=20.13, RMSE=3.86, MAPE=13.69) and Leave One Week One User Instance Out (MAE=3.12, MSE=20.14, RMSE=4.48, MAPE=7.16). Having the ability to predict change within an error margin of –3 to +3 can not only help in determining change in score but also aid in discerning change in levels of depression.

**Figure 8:**
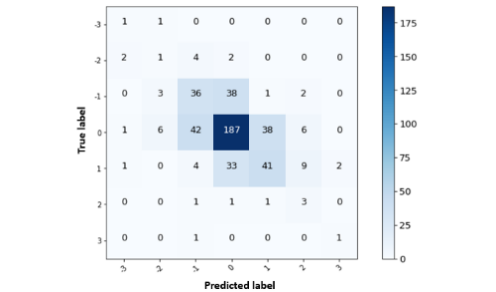
Confusion matrix for change in depression level into 7 classes (−3, −2, –1, 0, 1, 2, and 3) that represent transitions between higher and lower levels of depression.

We used the change in depression predictions to create 7 different classes marking changes in levels of depression [45]. The classes (−3, −2, −1, 0, 1, 2, and 3) map the regressed change in depression score to the change in depression level. The signs of the classes represent the rise and fall of depression level, and their values represent the magnitude of change in depression level. Similar to our approach to understanding how depression scores can be interpreted in terms of depression levels, this enabled us to visualize how well our models performed in terms of detecting change in depression score and mapping it to change in depression level. The results from the confusion matrix Figure 8 allowed us to see that the model was able to predict the level jumps (−1, 0, and 1) more accurately than the higher jumps (−3, −2, 2, and 3). This can be explained by the distribution of the observations in the data. Most of the recorded cases witnessed a rise and fall in depression levels by 1 or were at the same level (0) for an extended period. The confusion matrix showed that the true occurrences of large depression level jumps were very rare events.

**Table 3:** Depression score change regression results^a^.

	LOPO^b^	LWXO^c^	ACCU^d^	LOWOU^e^
MAE^f^ (SD)	3.28 (0.70)	3.24 (0.67)	3.21 (0.20)	3.12 (0.15)
MSE^g^ (SD)	21.35 (0.72)	19.43 (0.63)	20.13 (0.24)	20.14 (0.22)
MAPE^h^ (SD)	8.33 (0.55)	15.79 (0.61)	13.69 (0.17)	7.16 (0.20)
RMSE^i^ (SD)	4.2 (0.71)	4.26 (0.66)	3.86 (0.18)	4.48 (0.21)
Feature set	Fitbit, calls, conversation, screen, location, and Wi-Fi	Calls, conversation, screen, location, and Wi-Fi	Fitbit, calls, and location	Fitbit, calls, conversation, screen, and location
ML^j^ algorithm	AdaBoost	Random forest	XGBoost	Random forest

^a^The values presented display evaluation metrics for depression score regression models. The best-performing machine learning models were AdaBoost, random forest, and XGBoost.

^b^LOPO: Leave One Participant Out.

^c^LWXO: Leave Week X Out.

^d^ACCU: Accumulate Weeks.

^e^LOWOU: Leave One Week One User Instance Out.

^f^MAE: mean absolute error.

^g^MSE: mean squared error.

^h^MAPE: mean absolute percentage error.

^i^RMSE: root mean squared error.

^j^ML: machine learning.

### Feature Importance Calculation

One of the main advantages of modeling and formulating prediction strategies by extracting features using tree-based approaches is interpretability. In this section, we share the results and provide key insights into the features that were most influential in formulating our ML models. In particular, we chose our depression score prediction results to understand and narrow down the features that played a crucial role in model performance. The results are presented in two parts: (1) plot of the features from the best models based on the frequency of their selection and (2) analysis of the top 10 features with relative importance plotted for each of the personalized and universal modeling strategies.

### Most Frequent Features Selected During ML Modeling

Location, calls, and screen were the top 3 feature sets over all modeling strategies. The normalized location entropy and location entropy, which tell us how much time a participant spent at a location, were observed to be most frequently selected during modeling, in particular for both personalized and universal models. The other most frequently selected location features included the outlier time percentage, which is the ratio of time spent in a nonsignificant location divided by the time spent in all locations. Static ratio and number of location transitions were more features that were consistently included in the modeling strategies. Call features were the second most frequent feature in the model. Top call-related features included outgoing calls, in particular Shannon entropy for the duration of all calls, and minimum and mean duration of calls. This was also commensurate with the incoming call features, where, besides the mean, minimum duration of calls also included incoming call count and sum of duration of incoming calls. Frequently selected screen features included first use after unlock, count episode of unlocks, and minimum and maximum duration of screen unlocked. For completeness, we should also mention that conversation, Fitbit, and Wi-Fi followed the aforementioned feature sets.

Figures S1-S4 in Multimedia Appendix 1 show the features that were selected most frequently by Accumulate Weeks, Leave One Week One User Instance Out, Leave Week X Out, and Leave One Participant Out for the best models predicted under them. These plots show the number of times particular features were associated with the best models for all combinations of feature sets under the respective modeling strategy. It is important to note that we modeled up to 6 combinations of sensors and feature sets; therefore, the presence of a feature with a count of 6 indicates that, for all feature combinations tested, that particular feature played a significant role in predictive model building.

### Important Features Selected Based on Relative Importance From the Best Depression Score Prediction Models

In the previous section, we presented the results for the most frequently observed features that contributed to the modeling phase. In this section, Figures S5 and S6 in Multimedia Appendix 1 look at the feature importance of the modeling strategies. In particular, we will look at the relative importance among the top 10 features that influenced the respective modeling strategy. Relative importance reflects the importance that the ML algorithm places on a particular feature to form its predictions. Figure S5 in Multimedia Appendix 1 illustrates the feature importance for the Accumulate Weeks (left) and Leave One Week One User Instance Out (right) modeling strategies for depression score prediction. The feature set for Accumulate Weeks that performed best included Fitbit, calls, screen, and location. We see that *screen first unlock* has the maximum importance (0.175), followed by *screen maximum duration unlock* (0.115). They are followed by call features *count of most frequent call types* (0.0754) and *incoming call count* (0.0752).

In the case of Leave One Week One User Instance Out, the 6-sensor combination of Fitbit, calls, conversation, screen, Wi-Fi, and location performed best overall. The best features in this modeling strategy also included *screen first unlock* (0.16) and *screen maximum duration unlock* (0.112). This was followed by *call incoming count of most frequent call types* (0.079) and *screen count episode unlocks* (0.052). An important observation in this result is the similarity of the feature importance of both personalized models. Both modeling strategies selected screen, call, and Fitbit features as important. The results only showed the top 10 features by relative importance; other features such as location followed but had low relative importance.

The universal models are shown in Figure S6 in Multimedia Appendix 1. Both Leave One Participant Out and Leave Week X Out showed the best performance for 5-feature and 6-feature combinations, respectively. We note that both of the generalized approaches ranked the Wi-Fi feature *count of the most scanned access point for a time segment* with high importance (Leave One Participant Out: 0.11; Leave Week X Out: 0.132). Universal models also displayed importance among the screen and Fitbit features. Screen features such as *max duration unlock* (Leave One Participant Out: 0.042; Leave Week X Out: 0.07) and *standard deviation of duration screen unlocked* (Leave One Participant Out: 0.041; Leave Week X Out: 0.038) were also common between the 2 strategies. The Fitbit features of maximum resting heart rate and maximum steps were both selected as important by Leave One Participant Out and Leave Week X Out.

### Variation in Accuracy With Increase in Weeks of Data for Accumulated Modeling Strategy

**Figure 9:**
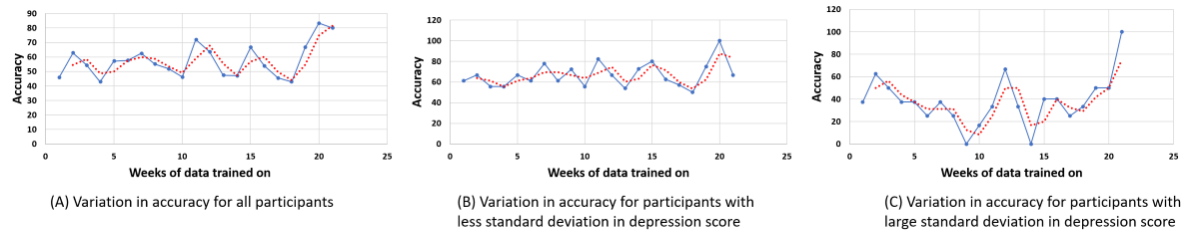
Variation in accuracy with increase in weeks of data trained on with a 2-point moving average to map the trend.

In this section, we present our analysis of how incremental increases in weeks of data affected model accuracy, which was extracted by converting depression score predictions to levels. The results in this section are based on modeling done under the Accumulate Weeks approach. We present 3 plots in Figure 9. The first plot reflects the variation of accuracy based on data from all participants, the second is for participants who showed low SD between their weekly PHQ-9 scores, and the final plot is for participants with high SD between their reported weekly PHQ-9 scores. The blue line represents the accuracy values corresponding to the weeks of data available for modeling, and the red dotted line represents a 2-point moving average.

All 3 plots in Figure 9 show that average accuracy fluctuated between weeks of data available for modeling. Therefore, we looked closely at the trend of the moving average to guide inferences about accuracy variation. In the plot with all participants, looking at the 2-point moving average, we can see that the average accuracy fluctuates between 50% and 60%. The plot with data from participants with a low SD in the PHQ-9 score is higher and can be conservatively stated to be between 60% and 75%. For participants with a high SD in the PHQ-9 score, the moving average shows a significant variation between 20% and 40% accuracy.

We observed that variation in the reported PHQ-9 score by participants contributed to the overall fluctuations. Ideally, it would be expected that, with an increase in data, the accuracy of the models would tend to increase. Although a slight trend upward was observed, the constant rise and fall of participant PHQ-9 scores seemed to affect the accuracy.

### Missingness of Data and the Impact on Accuracy and RMSE

**Figure 10:**
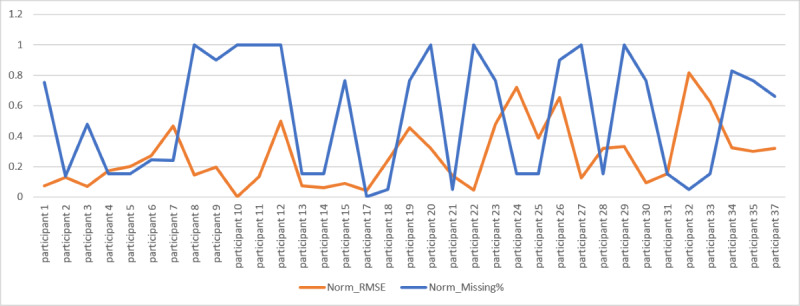
Missing data percentage (missing%) versus depression score prediction root mean squared error (RMSE)-normalized.

In this section, we explore how missing data affected our accuracy and RMSE values across participants. Figure 10 plots the percentage of missing data for each participant and their accuracy based on personalized modeling (Accumulate Weeks).

We observed 30.89% missing data in the phone-based sensors and nearly 67.74% in Fitbit. Missing Fitbit data were attributed to less than expected adherence to wearing the Fitbit because of reasons relating to regular charging, rash in some participants’ cases, and forgetting to wear the device on a regular basis.

The line in blue in Figure 10 is the normalized missing percentage, and the orange line is the normalized RMSE of predicting the depression score. The figure shows how missing data percentage relates to the RMSE value of individual participants for predicting depression score. To analyze both of these values, we normalized them to have the same scale of comparison. Observing a few participants, such as participant 22 (normalized missing percentage: 1; normalized RMSE: 0.04) and participant 24 (normalized missing percentage: 0.15; normalized RMSE: 0.72), we discovered the presence of an inverse relationship between model performance and the amount of missing data.

## Discussion

### Principal Findings

This study presented an in-depth analysis of passively sensed multimodal data collected over a period of 24 weeks from 37 adolescents to predict depression. The collection of data coincided with the COVID-19 outbreak and allowed for the observation of sensor data predictive capability in this scenario. Our models predicted both depression scores and change in the level of depression over weeks. The results showed reasonable improvements compared with the baseline models for both depression score and change in depression level prediction.

We explored universal and personalized modeling strategies. Overall, given the unpredictability of mental health patterns in individuals, personalized models were the most optimal. The Accumulate Weeks modeling approach, which relied on previous windows of sensor observations, achieved an RMSE of 2.83 for depression score predictions and an RMSE of 3.21 for change in depression score prediction. This provides a strong intuition regarding the model’s performance. In cases of depression prediction, the model can differ by a score of approximately 2 and, for change in depression score, by a score of approximately 3. This realization of the results points toward the future research and development of more sophisticated personalized predictive modeling to map individual behavioral traits between participants.

Investigating the modeling predictions by segmenting them into depression levels revealed that the model was good at predicting the mild, moderate, and moderately severe levels, whereas minimal and severe levels were difficult to detect because of the less frequent observations in the data collected. In the case of change in depression levels, the models detected decreases and increases with reasonable accuracy when the transitions were −1, 0, and 1. Rare changes such as −3, −2, 2, and 3 were detected with less accuracy. Data imbalance in terms of rare events such as severe changes in depression level, as shown in this study, can be a subject of further exploration, with possible strategies for synthetic data generation that can imitate sensor readings of participants with sudden or rare changes.

Our study also looked into feature frequency and feature importance. Understanding the features that were selected and highly ranked by optimal-performing models can help in determining what sensors to focus on when analyzing data from passive sensor studies. We see that location, calls, and screen sensor-based features appeared most frequently in the optimal-performing models. Individuals experiencing depressive symptoms tend to move less, which can be captured by location data. Depression also causes participants to reduce their interaction with friends and family, and call-related features can play a role in characterizing this behavior. The screen time of individuals has been seen to be a reflection of mood, as explored in an earlier study [48]. Feature importance helps narrow down the exact features that contributed to modeling. We noticed that, for personalized models, screen time was a strong determining factor that could be a consequence of the COVID-19 lockdown that prompted participants to use their phones with greater frequency. The feature importance presented in this study enabled us to make informed interpretable associations between sensor readings during changes in depression levels or scores. This can propel more research in the direction of more explainable or interpretable model building, especially for mental health–related diseases.

Personalized models performed best in our study of adolescent depression data. Therefore, it was important to understand how personalized modeling, in particular the Accumulate Weeks approach, performed when subjected to incremental data addition as well as looking at how missing values affected model performances. The Accumulate Weeks modeling approach performed better when the variation in the depression scores of the participants was low. By contrast, when the variation in depression scores was high, the accuracy decreased significantly. Exploring the relationship between missing values and the performance metrics of the models allowed us to discover an inverse relationship. This bolsters our understanding that completeness of data can be an important factor in improving model performance. Our experimental analysis of missing data suggests a requirement for strategies to improve the collection of sensor data that can include stronger adherence to protocols by participants or more robust data-generating processes.

Finally, we investigated how autoregressive integrated moving average (ARIMA) models performed in comparison with the ML modeling approaches used. The outcome of the comparison showed that ML models performed relatively better than the classic ARIMA models. However, the ARIMA models were more robust to sudden changes in comparison with the ML models, which were better at predicting smoother transitions. This result leads us to believe that, with possibly larger data sets, a combination of classic time-series approaches and ML-based approaches can be useful for participants with inherent trends or seasonality in their behavior.

### Comparison With Previous Research

In the *Related Work* section, we discussed a number of studies on mobile health. In particular, we looked at studies that explored adolescent depression to some degree. Most previous studies were conducted with varied age groups where the adolescent population was either not a part or a very small part of the study [33-38]. However, these studies cannot be considered representative of the adolescent age category. An in-depth review of passive sensing technology for predicting depression [49] mostly focused on college students and adults.

This study focused on adolescents, and all the results are representative of adolescent participants with a previous diagnosis of depression. To the best of our knowledge, this study had the largest sample of adolescents monitored passively to predict depression. In our work, we found location to be one of the most frequent feature sets to be recognized by the ML models. This is in agreement with previous studies of GPS sensor data [33,34] to detect depressive states. They too found a relationship between mobility metrics and depression. The population segment in those studies was restricted to adults. The sensors used were also limited. Other studies that used multimodal sensor data were limited in either participant recruitment or duration [33-37] as well as the population they studied. Our study was an extensive, 24-week–long endeavor with 37 participants being retained for our predictive analysis. A few previous studies on the adolescent population relied on survey-based approaches [40-42]. We differ in relation to them as we strictly based our modeling on multimodal sensor features and did not rely on any direct input from the participants or their parents.

The work by Cao et al [39] was the closest to our study. It was aimed at the adolescent population and used a combination of survey inputs from parents and adolescents besides multimodal features to improve on their accuracy. The differences between the study by Cao et al [39] and our study lie in the type of modeling approaches we used (eg, universal and personalized), the duration of the trial, and the number of participants. In relation to the modeling approaches, Cao et al [39] only performed a universal approach with the best RMSE value leading to 3.70, which combined parents’ inputs, steps, GPS, SMS text messages, and calls. We achieved an RMSE of 2.39 based only on the sensor combinations of calls, screen, location, and Fitbit. Their trial lasted 8 weeks and only had 8 participants; this was less compared with our study. Overall, our study explored adolescent depression based on passively sensed data in greater depth owing to both modeling approaches, the study duration, and the participants involved. We also showed how data affected our modeling and compared them with classic techniques such as ARIMA. This information is pertinent in understanding the adolescent population and provides evidence of the type of modeling approaches and features that can generate the best results in predicting depression and change in depression score.

### Limitations

Despite the exhaustive modeling approach and strong participant involvement, our study also encountered some limitations. One of the primary limitations of our study was the start of the COVID-19 outbreak that caused potential deviation from the regular behavior of adolescents. Schools were closed and mobility was restricted to the confines of participant houses or rare outings. Most adolescents were at home and were restricted to television, games, or cell phone use.

We also encountered missing data partially because of the participants’ lack of adherence to data syncing and management of the app and partially because of technical issues and difficulty in remote troubleshooting. Missing data are a general concern in passive sensing, which we investigated in our work to show the impact they had on modeling. Data completeness can aid greatly in modeling performance.

Despite our study being one of the studies of longest duration conducted to the best of our knowledge, it can still be categorized as a small data set. A small data set can have an impact on the modeling of rare events; for example, large, sudden jumps in depression scores or extreme depression scores can be hard to track for ML models. Although these measures are anomalies in the data set, perhaps a more focused study on participants exhibiting such traits can be looked into for future directions.

Finally, although our extensive analysis provides useful insights into the feasibility and challenges of using passive sensing for the prediction of adolescents’ depression, we emphasize that our study is exploratory and further investigation and more studies are needed to replicate these results.

### Conclusions and Future Directions

In this exploratory study, we investigated the feasibility of using passively sensed data for predicting adolescents’ depression. We applied universal and personalized ML approaches to predict depression score and change in depression level in adolescents. Our results showed RMSE values of approximately 2 and 3 for the prediction of depression score and for depression change, respectively. This provides confidence in personalized modeling approaches for predicting depression in adolescents. We also investigated the features that models frequently relied on. Features related to screen, call, and location sensors were the most frequent in the optimal models. Our analysis showed better model performance for participants with low variation in depression scores. We also observed that the percentage of missing data of a participant inversely affected the model’s performance.

Modeling both change in depression and depression scores can be greatly influential in helping clinicians, parents, and adolescents take preventive measures to intervene in the early worsening of depressive symptoms before entering severe categories. This study will inform the development of an adolescent-facing mobile app with a parent and clinician component to aid in adolescents’ self-management and tracking of their mood.

Future research based on our principal findings can help improve mental health prediction. The area of personalized modeling can be used to provide tailored feedback to patients. Rare event prediction in the face of the data imbalance seen in this study should act as an impetus to develop more realistic synthetic data. The feature importance determined can be further explored for other mental illnesses and provide a more interpretable analysis of passive sensing–based studies. Strategies to mitigate missing data for passive sensing will need to balance both participant adherence and modeling strategies that account for missingness. A final promising area of research could be the formulation of an ML model while incorporating a classic time-series approach to account for possible future trends in patients.

## Appendix

Multimedia Appendix 1 Feature importance.

## Abbreviations

ARIMA: autoregressive integrated moving average
CV: cross-validation
EDA: exploratory data analysis
MAE: mean absolute error
MAPE: mean absolute percentage error
ML: machine learning
MSE: mean squared error
PHQ-9: Patient Health Questionnaire-9
RMSE: root mean squared error
