# Supplementary material for: Predicting Depression in Adolescents Using Mobile and Wearable Sensors: Multimodal Machine Learning–Based Exploratory Study
Source: JMIR Form Res. 2022 Jun 24;6(6):e35807. doi: 10.2196/35807 (PMC9270714; doi:10.2196/35807)
Supplement: Multimedia Appendix 1 [file formative_v6i6e35807_app1.doc]

## **Supplemental Section**

#### ***Most Frequent Features Selected During ML Modeling Plots and Additional explanation***

As mentioned in the results section, Calls, Location and Screen were the most frequent feature sets selected during modeling. Figures S1 to S4 present this information in a graphical format. Due to the large number of features under each of the feature sets we focus our attention to the most frequent of features within these 3 feature sets. The most frequent features are associated with location. The normalized location entropy and location entropy which tells us how much time a participant spent at a location is observed to be most frequently selected during modeling in particular for both personalized and universal models. Other location-based features that have been most frequently observed are the outlier time percent which is the ratio of time spent in a non significant location divided by the time spent in all locations. Moving to static ratio and number of location transitions are more features that have consistently featured in the modeling strategies.

Call features have been the second most frequent features in the modeling. Top call related features include outgoing calls in particular Shannon entropy for the duration of all calls, minimum and mean duration of calls. This is also commensurate with the incoming calls features, which besides mean, minimum durations of calls also include incoming call count and sum of duration of incoming calls.

Screen related features have been detected too and have been more prominent than other feature sets. Screen related features include first use after unlock, count episode of unlocks, minimum and maximum duration of screen unlocked. Overall location, calls and screen features have contributed to most of the best models. For completeness, we should also mention that conversation, fitbit, WiFi followed respectively the aforementioned feature sets.


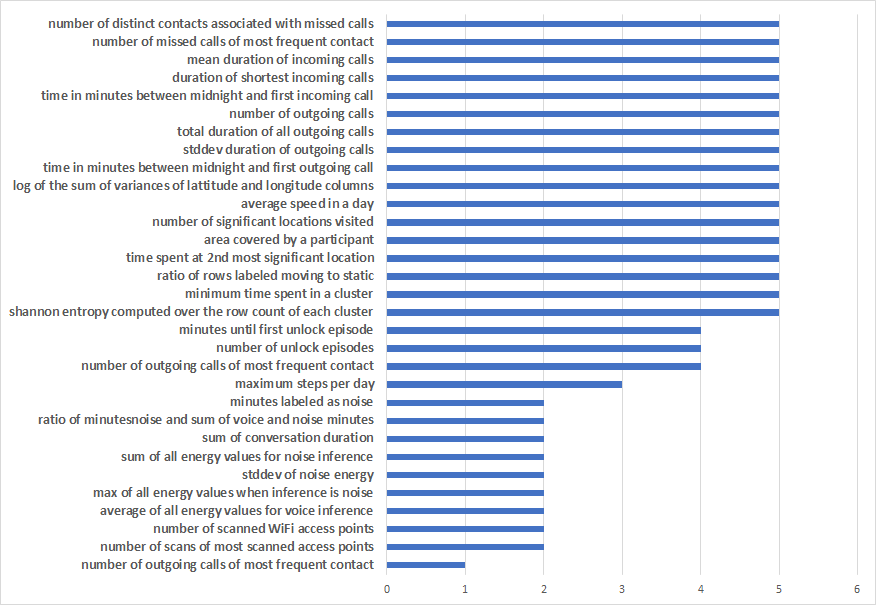


Figure S1: Frequency of features from the best models with Accu modeling strategy for depression score prediction


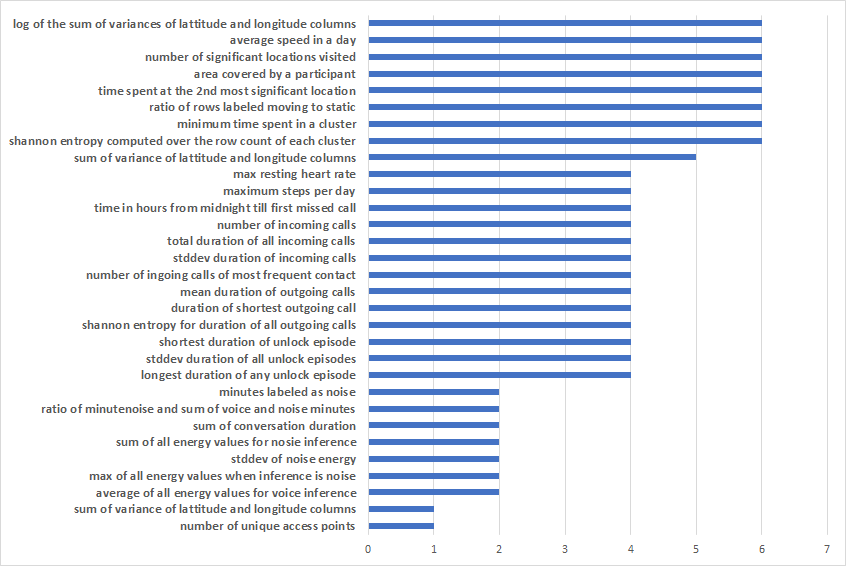


Figure S2: Frequency of features from the best models with LOWOU modeling strategy for depression score prediction


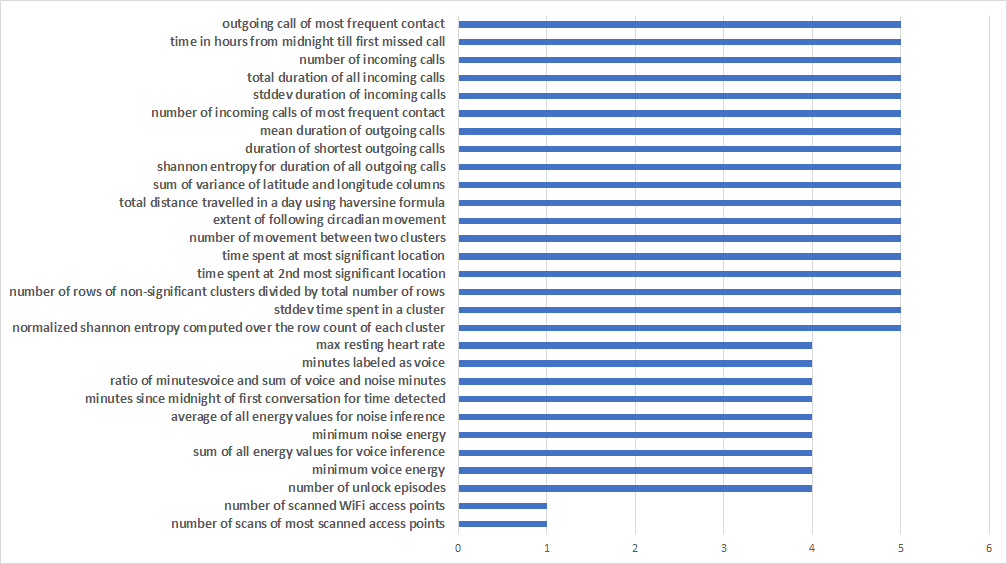


Figure S3: Frequency of features from the best models with LOPO modeling strategy for depression score prediction


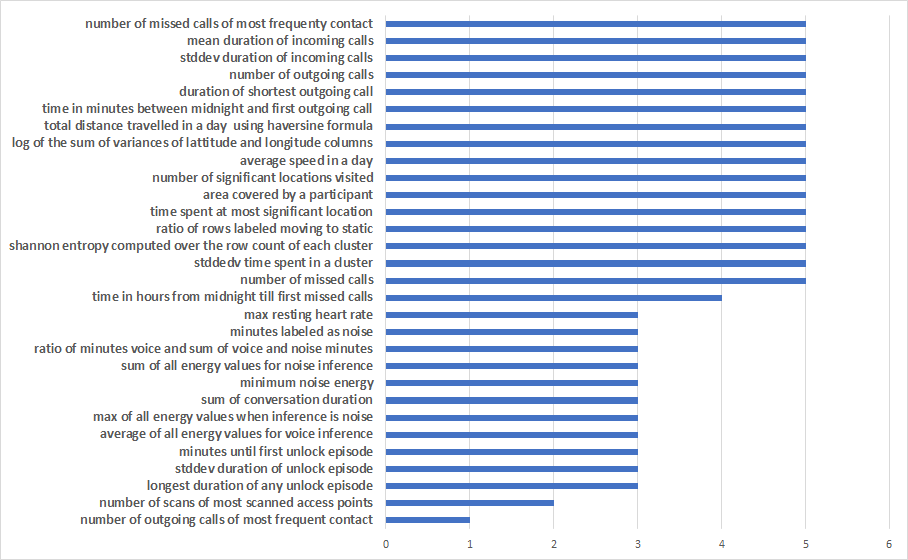


Figure S4: Frequency of features from the best models with LWXO modeling strategy for depression score p

#### ***Important features selected based on relative importance from the best depression score prediction models***


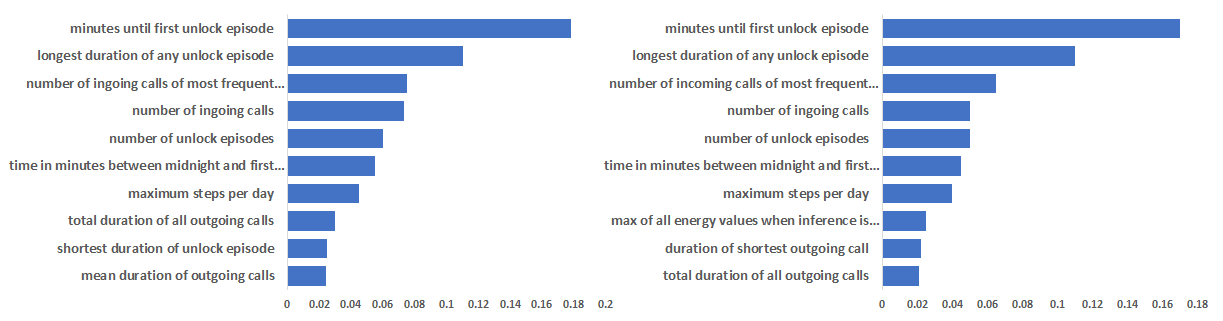


FigureS5: Feature importance based on best model for ACCU (left) and LOWOU (right) for depression score prediction


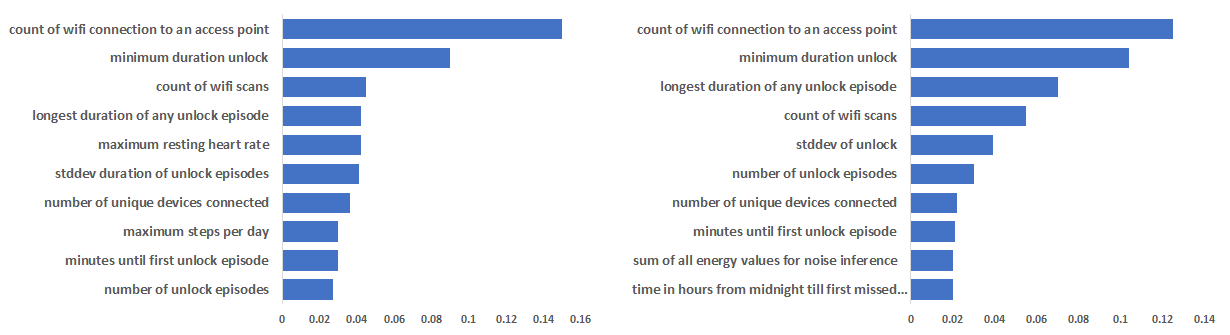


Figure S6: Feature importance based on best model for LOPO (left) and LWXO(right) for depression score prediction
